# Determination of Xanthohumol in Hops, Food Supplements and Beers by HPLC

**DOI:** 10.3390/foods8100435

**Published:** 2019-09-24

**Authors:** Patricia Vázquez Loureiro, Ignacio Hernández Jiménez, Raquel Sendón, Ana Rodriguez-Bernaldo de Quirós, Letricia Barbosa-Pereira

**Affiliations:** Department of Analytical Chemistry, Nutrition and Food Science, Faculty of Pharmacy, University of Santiago de Compostela (Spain), 15782 Santiago de Compostela, Spain; patriciavazquez.loureiro@usc.es (P.V.L.); nachoheji@gmail.com (I.H.J.); ana.rodriguez.bernaldo@usc.es (A.R.-B.d.Q.)

**Keywords:** xanthohumol, dietary supplements, HPLC-DAD-MS/MS, method validation, food analysis and composition, dose, nutritional intake

## Abstract

Xanthohumol (XN) is the main prenylated chalcone present in hops (*Humulus lupulus*) with high biological activity, and it is of great importance for human health because of its antioxidant, anti-inflammatory, immunosuppressive and chemopreventive properties. This polyphenol can be included in the diet through foods in which hops are used, such as beer or food supplements. Because of their health benefits and the increasing interest of using hops as a novel nutraceutical, the aim of this work was the identification and quantification of XN in different types of samples using a method based on high resolution liquid chromatography with a diode array detector (HPLC–DAD). The method was validated in terms of linearity, limits of detection (LOD) and quantification (LOQ), repeatability and recovery. Acceptable linearity (*r*^2^ 0.9999), adequate recovery (>90% in the most of cases) and good sensitivity (LOD 16 µg/L) were obtained. Furthermore, the presence of XN in all samples was confirmed using liquid chromatography coupled to mass spectrometry (LC-MS/MS) operated in negative ESI (electrospray system ionization) mode. The concentrations of XN determined in hop flowers and food supplements were above the LOQ, in a range between 0.106 and 12.7 mg/g. Beer may also represent an important source of dietary prenylflavonoids, with between 0.028 and 0.062 mg/L of XN. The results showed that the methodology proposed was suitable for the determination of XN in the different types of samples studied, and the amounts of XN varied significantly according to the selected product.

## 1. Introduction

The beneficial effects of a moderate consumption of beer are associated to the bioactive compounds present in the beverage. Xanthohumol in particular, has attracted the attention of the scientists, thanks to is biological effects [1,2].

**Figure 1:**
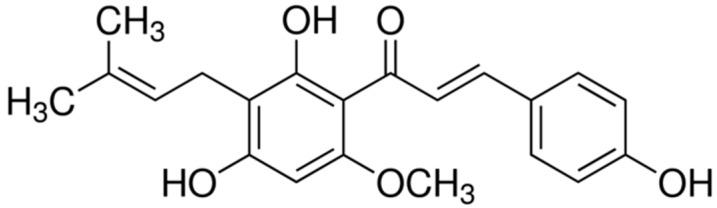
Structure of xanthohumol.

Xanthohumol (3′-[3,3-dimethyl allyl]-2′,4′,4-trihydroxy-6′-methoxychalcone) (Figure 1) is the main prenylated flavonoid of the female inflorescences of the hop plant (‘hops’), an ingredient of beer.

Different biological activities have been attributed to prenylflavonoids from hops, such as prevention or treatment of (post-)menopausal ‘hot flashes’ and osteoporosis; treatment of excitability and restlessness associated to tension headache; the ability to whet one’s appetite and to improve digestion; relief for toothaches, earaches and neuralgia; and anticancer properties [3,4]. The female inflorescences of *Humulus lupulus* (hops) have been also used in traditional medicine mainly to treat sleep disturbances and in brewing industry to increase the bitterness and aroma [4]. The traditional use of *Humulus lupulus* flowers for the relief of mild symptoms of mental stress and insomnia was also reported by the Committee on Herbal Medicinal Products (HMPC) of the European Medicines Agency (EMEA) (2007). Furthermore, the German Commission E and European Scientific Cooperative on Phytotherapy approved hops for the treatment of excitability, sleep disturbances, and others [5,6].

Furthermore, xanthohumol (XN) was suggested by Hirata et al. (2017) [7] to be antiatherogenic, since it increases high-density lipoprotein (HDL) cholesterol levels.

The prenylated flavonoid also exhibits anti-inflammatory properties, and additionally, recent studies have revealed that this compound inhibits HIV-1 [8,9].

With respect to the antioxidant properties, in assays based on the capacity to inhibit the oxidation of LDL in vitro, XN exhibited a strong antioxidant activity, higher when compared with α-tocopherol but lower compared to quercetin [1].

In addition to their well-known healthy effects, commented on above, hop polyphenols can contribute to beer’s organoleptic properties, particularly bitterness and astringency. This effect depends on their degree of polymerization [10].

On the other hand, the flowers of *Humulus lupulus* are a natural source of food flavoring for cereals, spices, sauces, tobacco and alcoholic beverages other than beer [11]. Hops were also used in perfumes, especially the spicy and oriental types, and in skin creams and lotions [12].

Although beer is the main dietary source of XN, the XN levels in commercial beers are relatively low, thus some studies have reported values around a maximum of 15 mg/L. This can be explained by the fact that the XN is isomerized to isoxanthohumol (IXN) during wort boiling. Contradicting 15 mg/L, high concentrations of IXN were found as 0.04 to 3.44 mg/L in [1,3]. However, it is interesting to note that nowadays, many supplements containing the phenolic compound are commercialized [13].

From the analytical point of view, several analytical methodologies and extraction procedures to determine XN and prenylflavonoids have been reported in the literature. Liquid chromatography with diode array detection or coupled to mass spectrometry has been widely used to quantity the active compounds. Regarding the extraction process, conventional solid-liquid methods and more sophisticated approaches that involve the use of supercritical fluids or high pressure treatments. An overview of the main techniques and extraction methods is summarized in Table 1.

The composition analysis of XN in dietary supplements is limited, since almost all the methodologies reported in the literature have been employed on beer and hop. Moreover, method validation for different food matrices and their application to a wide range of samples, including dietary supplements, have not been considered in previous studies.

This study aims to determine XN in hops, food supplements and beers, and for that purpose an HPLC-diode array detector (DAD) method was used. The method was validated in terms of linearity, limits of detection and quantification, repeatability and recovery. Additionally, LC-MS/MS operated in negative electrospray system ionization (ESI) mode was used as a confirmatory technique.

## 2. Materials and Methods

### 2.1. Chemicals and Standards

Methanol and acetonitrile, HPLC grade, and acetonitrile, hypergrade, for LC-MS were supplied by Merck (Darmstadt, Germany). Formic acid, HPLC grade, purity 50%, was supplied by Fluka analytical (St. Louis, MO, USA). Xanthohumol (CAS number: (6754-58-1), purity 98%) was purchased from Carbosynth (Berkshire, UK). Purified water (Type I) was obtained from an Autwomatic purification system (Wasserlab, Navarra, Spain).

An individual stock solution of xanthohumol was prepared in methanol at the concentration of 1 mg/mL. Intermediate standard solutions were prepared in methanol by diluting the stock solution in the concentration range 0.1–20 mg/L; a solution of 0.05 mg/L and 0.016 mg/L were prepared to determine the limits of detection and quantification respectively. Stock and working standard solutions were stored at 4 °C and protected from the light before analysis.

### 2.2. Samples

Five different commercial food supplements in the dose form of capsules (A, D, G, M and N) containing extracts of hops were purchased from a local pharmacy. The main ingredients listed on each product label are shown in Table 2. The flowers of hop (hops, H) were purchased in a local herbalist’s shop. Five different types of beers, described in Table 3, were purchased in a local supermarket.

### 2.3. Samples Preparation

For analysis, three hops and three capsules of each food supplement were randomly selected and homogenized. Then, 100 mg of sample was weighed and extracted with 10 mL of methanol by sonication for 30 min at 25 °C using an ultrasonic bath (Branson 5510, Branson Ultrasonic Corp., Danbury, CT, USA). The extracts yielded were diluted (when required) and filtered through a 0.22 µm PTFE membrane filters before HPLC analysis. For beer analyses, samples were first degassed by sonication for 1 h. Then, 10 mL of each sample was loaded on a C18 solid-phase extraction (SPE) cartridge (Waters Corporation, Dublin, Ireland) after conditioning with 5 mL methanol and 5 mL water. Next, the SPE cartridge was washed with 5 mL of water and the fraction of interest was eluted with 5 mL of methanol. A portion of the extract was filtered with a 0.22 µm PTFE membrane filter before HPLC injection. Fractions yielded from samples G and MS were evaporated to dryness under a stream of nitrogen at 40 °C, using an evaporator system Labconco Rapid Vertex-Evaporator (Labconco Corporation, Kansas City, MO, USA) to concentrate ten times, and fractions yielded from samples M and B were concentrated 2 times under the same procedure. The residues were finally redissolved in methanol and filtered through 0.22 µm PTFE membrane filters for further HPLC analysis. All determinations were performed in triplicate.

### 2.4. HPLC-DAD/UV Analysis

The identification and quantification of XN was performed with a high-performance liquid chromatography (HPLC) system, model 1100 HP (Hewlett-Packard, Waldbronn, Germany) equipped with vacuum degasser, a quaternary pump, an autosampler, a column thermostat system, and a diode array detector (DAD), controlled by HP ChemStation software (version B.03.0.1). Separation was performed on a reverse phase C18 Kromaphase 100 (150 × 3 mm, 5 µm size of particle) column from Scharlau (Barcelona, Spain), thermostated at 30 °C. Two solvents were used as mobile phases: water 0.1% formic acid (v/v) (solvent A) and acetonitrile 0.1% formic acid (*v*/*v*) (solvent B). A gradient-elution was applied as follows: 80% A, 0–3 min 20%–50% B, 3–6 min 70% B, 6–15 min 100% B, 15–20 min 100% B, 20–25 min 100%–20% B. The injection volume was 20 µL and the flow rate was 0.5 mL/min. Scanning was performed continuously at wavelengths between 200 and 400 nm. The XN was identified by comparison of the retention time and UV spectra with that obtained with pure standard injected under the same chromatographic conditions. Quantifications were carried out by an external standard method with a nine-point calibration curve at 370 nm.

### 2.5. LC-MS/MS-ESI Analysis

An LC-MS/MS system comprised of an Accela autosampler, a column oven and an Accela 1250 pump fitted with a degasser, coupled to a triple quadrupole mass spectrometer TSQ Quantum Access Max controlled by Xcalibur 2.1 software (Thermo Fisher Scientific, San José, CA, USA) was used to confirm the identity of XN. The chromatographic conditions were similar to those of the HPLC analysis described above. The mass spectrometer operated in negative ESI (electrospray system ionization) mode. The optimized MS/MS detector settings were as follows: spray voltage 2.5 kV; tube lens voltage was −99 V; vaporizer and capillary temperatures were set at 340 °C and 350 °C, respectively; nitrogen was used as sheath gas (pressure 35 psi) and as auxiliary gas (pressure 10 arbitrary units); and Argon was used as the collision gas (1.5 mTorr). MS data were acquired in selected reaction monitoring (SRM) mode. Besides the retention time, the precursor ion and the fragmentation ions obtained and the ratio between them were used as parameters to confirm the presence of XN in the entire set of samples analyzed.

### 2.6. HPLC-DAD/UV Method Validation

The HPLC-DAD method was validated for the following parameters: sensitivity, linearity, precision and accuracy. The limits of detection (LOD) and quantification (LOQ) were determined experimentally by serial dilutions of XN standard necessary to reach signal-to-noise ratios of 3 and 10, respectively. To evaluate the linearity (*r*^2^), a calibration curve with nine points 0.5–20 mg/L of XN were analyzed in triplicate for each concentration level. The precision of chromatographic method was evaluated through the multiple injection of the several standard curve levels (0.05–20 mg/L) intra-day for repeatability and inter-day for intermediate precision, and the percentages of relative standard deviation (RSD) values were determined for all data. The precision of the extraction was validated by repeating the extraction procedure of each sample. Recovery tests were performed to evaluate the accuracy of the analytical method. Food supplements A, D, G and M were spiked with aliquots of 100 µL of 100 mg/L, 250 mg/L and 500 mg/L standard solution depending on the spiked concentration. Additionally, for hops, 100 mg of sample was spiked with aliquots of 100 µL, 250 µL and 500 µL of 1000 mg/L stock solution, and was then extracted following the same procedure described above in Section 2.3.

For beer, samples B, M and E were spiked with aliquots of 100 µL of 5 mg/L, 10 mg/L and 50 mg/L standard solution, while samples G and MS were spiked with an aliquot of 100 µL of 2.5 mg/L, 5 mg/L and 10 mg/L standard solution, because of their lower concentrations of XN. The fortified samples were then extracted and analyzed as described above in Section 2.3. All experiments were performed in triplicate.

## 3. Results and Discussion

### 3.1. Method Validation HPLC-DAD

The HPLC-DAD method for Xanthohumol detection and quantification in food supplements, hops and in different types of beers was fully validated according to different parameters, such as linearity, sensitivity, precision, accuracy and recoveries under optimal conditions. The results are shown in Table 4, Table 5 and Table 6.

Linear regression analysis was used to quantify XN in the samples. Nine standards solutions of known concentrations between 0.05 mg/L and 20 mg/L were used to obtain the calibration line by representing the peak area of each one at the maximum XN wavelength of 370 nm against the known concentration. This range was selected according to the XN levels in samples. Appropriate linearity was obtained within the range of concentrations studied with a correlation coefficient of 0.9999, as shown in Table 4. This *r*^2^ value was better than others described in the literature for the analysis of XN (0.986–0.9993) using HPLC-DAD or UHPLC-PDA [13,14].

The limit of detection (LOD) was determined as three times the signal-to-noise ratio and the limit of quantification (LOQ) was determined as ten times the signal-to-noise ratio, according to the American Society ACS guideline [24]. The low LOD and LOQ values of 0.016 mg/L and 0.05 mg/L, respectively, confirm the good sensitivity of the method. These values were better than those described by Dhooghe et al. (2010) [13] for hop extracts using HPLC-DAD and were similar to those described by Bernal et al. [18], for the analysis of beer samples by HPLC-DAD (LOD 0.01 mg/L). Despite the good results, the values reported in some previous studies were slightly better than those obtained in our study. Chen et al. (2010) [17] reported LOD and LOQ values of 0.003 mg/L and 0.01 mg/L, respectively, for beer analyses using a methodology based on the coupling of cloud point extraction (CPE). Additionally, Gonçalves et al. (2013) [14] described lower values using MEPS_C18_/UHPLC-PDA (LOD and LOQ of 0.0009 mg/L and 0.003 mg/L, respectively).

Considering the specificity, the peaks of XN in the chromatograms were identified by comparing its UV spectrum and the retention time of those obtained with the reference standard.

**Figure 2:**
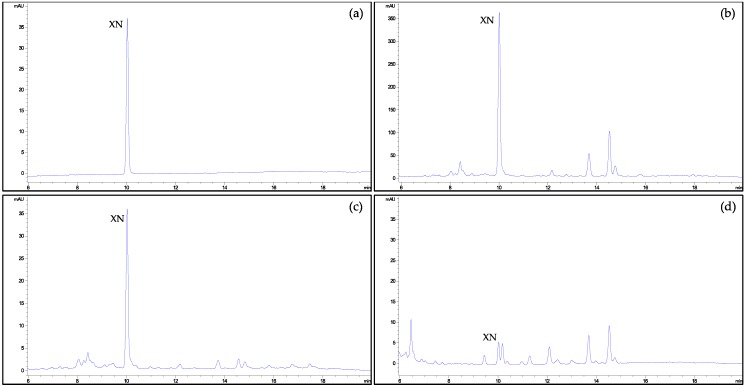
HPLC-DAD chromatograms monitored at 370 nm for xanthohumol (XN) determination: (**a**) standard of XN at 1 mg/L; (**b**) hops; (**c**) food supplement D; and (**d**) beer E.

The retention time RSD% values and the concentrations’ standard deviation values denoted an excellent precision of the chromatographic parameters of separation in terms of repeatability intra-daily and in terms of reproducibility inter-daily. Minimal changes in the operating conditions did not affect the chromatographic separation, confirming the robustness of the analytical procedure proposed. Moreover, no interferences were detected in the determination of XN from other ingredients present in the different food supplements, hops or beers, demonstrating the selectivity of the method (see chromatograms in Figure 2). For beers, a second compound eluted closely to the XN, although the integration was not significantly affected (Figure 2d).

The accuracy of the method was checked by recoveries assays spiking all the samples at three different levels of a known concentration of XN in triplicate. The concentration levels used in the recovery assays were adjusted considering the initial amount of XN present in samples. The procedure used was the same already described in the section of sample preparation, and the results of XN recoveries are shown in Table 5 and Table 6. Very good results were found for food supplements, with percentages of recovery values of XN ranging from 72.6 to 109.6% (Table 5). Very few studies have developed and validated analytical methods for the determination of XN in food supplements. Dhooghe et al. (2010) [13], described similar recovery values of XN for hop extract and derived capsules. In the case of beers, a good accuracy was observed for samples B, E and Mh, with recovery values ranging from 73.3 to 109.6% (Table 6). The accuracy was less satisfactory for beer samples S and MS, with recovery values lower than 53.9% for S, and for MS samples when spiked at the lower concentration of fortification (data not shown). Taking into account beers, more studies might be found in the literature related to validation methods for the determination of XN. Nevertheless, the validation was not performed for all types of beer. In this study, the validation was extended to other types of beers. The accuracy observed in this study was similar to that described in the literature for beer type: Pale lager (74.2%–99.9%) [14].

The good performance of the method was also elucidated by the precision results. The low values of RSD% indicated a satisfactory precision of the chromatographic parameters of the separation and extraction procedures, in terms of repeatability intra-daily, confirming the robustness of the proposed methodologies.

Values below 6% of relative standard deviation were obtained for the US-assisted extraction-HPLC-DAD used in food supplements and the SPE-HPLC-DAD method employed for beers. However, the precision was less satisfactory in samples spiked at the lower levels of concentration. Furthermore, high values of RSD% were also observed for samples D (food supplement) and Mh (beer). These results could be related to the type of sample or to the presence of some components that may interfere in the recovery of XN (see Table 5 and Table 6).

### 3.2. The Determination of XN in Samples by HPLC-DAD

The developed method was applied to identify and quantify XN in five food supplements, five beers and hops. The contents of XN in food supplements and hops, expressed as µg/capsule, are reported in Table 2. Concentrations of XN above the limit of quantification were detected in almost all the food supplements analyzed, except for sample G. The values of XN obtained showed a noteworthy variability among examined samples, ranging from 56.42 to 3602.34 µg/unit. Taking the examples of samples D and N, similar contents of hops extract were declared on their labels (about 30 mg/unit) and similar analytical contents of XN, around 57 µg/unit, were determined. On the other hand, the labels of samples G and M declared 100 mg of hops extract/unit but the concentration of XN found was not the same. Indeed, sample M was that with the highest concentration of XN among all supplement analyzed, while in sample G, the bioactive compound was not detected. These results could be related to the characteristics of the hops that were used to produce the extracts; the origin; or other parameters related to their production and treatment, such as the climate, the harvest and post-harvest processing, store conditions or other unknown factors. Another reason for these results could be the presence of other components in the capsules, such as isoflavones and evening primrose oil, which were mentioned on their labels, and may interfere in the extraction of XN. The low extraction yield could justify the lack of sensitivity when determining its content by HPLC-DAD, since it was below the limit of detection, even though the confirmation of XN by HPLC-MS/MS was achieved. Taking into account one unit/capsule of each supplement, the content of XN/capsule increases in the following order: G < D < N < A < M. However, considering the intake recommendations per day, the high intakes of XN will be achieved by consumption of the supplements as follows: G < N (57 µg/day) < A (up to 280 µg/day) < D (up to 396 µg/day) < M (3602 µg/day).

The methanolic extract of hops yielded in the present study contained 855.7 µg/g of XN that correspond to 0.86% of total weight of hops (see Table 2). According to Stevens et al. (2004) [3], the amounts of XN in hops can vary from 0.1% or less for aged hops to over 1% for high xanthohumol-producing varieties. Česlová et al. (2009) [16] analyzed hop extracts and reported XN values between 0.59 ± 1 and 107 ± 2 mg/L determined by HPLC, and between 0.37 ± 0.01 and 125 ± 3 mg/L using spectrometry analysis. The lowest concentration of XN was detected in extracts prepared by supercritical CO_2_ extraction and the highest concentration found of XN corresponded to the extract prepared by supercritical CO_2_ extraction using ethanol as a modifier.

Considering beer samples, the amounts of XN determined ranged between below the limit of detection and 61.55 µg/L (see Table 3). The Lager beer showed the highest concentration of XN, while craft and amber beers showed similar levels, 28.49 and 29.82 µg/L respectively. In the present study, non-alcohol beer displayed the lowest concentration of XN compared to lager, amber and craft beers, as described by other authors in the literature. For beer type stout, the values of XN obtained in this study were not in accordance with those described by Stevens et al. (1999). Those authors determined in imported stout beers, the highest concentration of XN compared to lager or pilsner beers. In several studies comparing different types of beer, the stout type was one with high amounts of XN, even higher than the lager type [3,14,25]. Our results could be attributed to the limitation of the cartridge of SPE used for the extraction, since this type of beer may contain different components in its matrix that may interfere with the elution of XN.

The results obtained were comparable with other studies, such as the Czech study performed by Česlová et al. (2009) [16] in which Czech beers were directly analyzed by HPLC-MS without pre-treatment. The concentrations of XN in the beers studied were found to be between not detectable and 0.09 mg/L in mass spectrometry, and not detectable in UV, since the limit of detection by HPLC was 0.02 mg/L and the limit of detection by HPLC-MS/MS was 0.006 mg/L. However, the XN values observed were lower than those described by Chen et al. (2010) [17] in beers from different regions of China. Those authors reported contents of XN in the range of 0.052 to 0.628 mg/L, using a method based on the coupling of cloud point extraction (CPE) with HPLC-UV. Stevens et al. (1999) [21], reported contents of XN between 0.002 mg/L in lager beer and 0.34 mg/L in stout beer and the differences observed may be due to the different producing areas of the beer samples.

The contents of XN in fresh beer samples from Germany were evaluated by Intelman et al. (2009) [26] in using the ECHO Technique. Values between 0.0028 and 0.137 mg/L were reported, and an increase in the concentration of XN during the storage of beer was observed. On the contrary, in the study carried out by Bernal et al. (2011) [18] using HPLC-DAD, XN was not detected in the beer samples analyzed. These authors analyzed dark beers, golden/pale beer and non-alcoholic beer, and in all cases the contents were below the limit of detection (0.01 mg/L).

### 3.3. Xanthohumol Identification by LC-MS/MS—Confirmatory Technique

LC-MS/MS was used to confirm the identity of XN in the different sets of food supplements, beers and hops. A methanolic standard solution was analyzed by direct infusion by use of a built-in syringe pump, to evaluate the MS-generated precursor ion and product ions of XN. Full scan data acquisition was performed within the range 100–400 m/z. The protonated molecular ion [M + H]^−^ corresponded to the more intensive precursor ion. The transitions used for XN were the following m/z: 353.2→119.1, 353.2→174.9 and 353.2→233.0; and the energy collisions used were −37 V, −35 V and −22 V, respectively. These results are consistent with those found by other authors [21,26]. The ratio between the two selected transitions was used for the verification of the identity of the XN in the samples. The ion ratio was 1.37 and the retention time 8.27 min. Selected multiple reaction monitoring transitions (MRM), retention time and adjusted voltage settings are shown in Table 7. The presence of XN was confirmed for all samples analyzed, including the samples with XN contents lower than LOQ with HPLC-DAD methodology.

## 4. Conclusions

Because of the numerous biological activities recognized to XN in numerous studies, the number of products and foods fortified with hops extracts has recently increased in the market.

Several developed methods for the determination of XN in beers are available in the literature. However, studies describing methodologies for the determination of this bioactive compound in food supplements are scare in the literature. In this study, an analytical methodology for the identification and quantification of XN was successfully achieved. The HPLC-DAD method showed excellent linearity, good precision and sensitivity to detect XN in a set of food supplements, hops and beers. The ultrasound assisted extraction-HPLC-DAD method for hops and food supplements, and the SPE-HPLC-DAD method for beers were the most appropriate for this type of compound. Nevertheless, the matrix effect must be taken into account in the case of stout beer samples, where the recoveries of XN were lower than those observed for the rest of samples.

The results confirmed that the contents of XN in the different food supplements analyzed were decidedly variable and underlined the need to control this type of products. The results also highlighted that the contents of XN in food supplements were significantly higher than those present in food products, such as the set of beer samples.

The methodology proposed in this study may be of great significance to verify the presence of XN, as one of the main bioactive compounds in the extracts of hops, to ensure the nutritional/dietary recommended intakes, and therefore, the beneficial effects on health declared on the food supplements labels.

## Figures and Tables

**Table 1:** An overview of the analytical techniques and extraction procedures for xanthohumol (XN) and prenylated flavonoids’ determination.

Sample	Analyte	Extraction Procedure	Analytical Method	Reference
Hop extract and capsules	XN, IXN, 8-PN and 6-PN	Extraction with methanol + ultrasonic bath	HPLC-DAD Mobile phase: (A) 0.25% formic acid in water and (B) 0.25% formic acid in acetonitrile Column: Lichrospher RP-18e (244 × 4 mm, 5 µm) λ 290, 370 nm	[13]
Beers	XN and IXN	Microextraction by packed sorbents (MEPS)	UHPLC-PDA Mobile phase: (A) 0.1% formic acid in water and (B) acetonitrile Column: Acquity UPLC^TM^ strength silica HSS T3 (100 × 2.1 mm, 1.7 µm) λ 290, 288, 368 nm	[14]
Hop products	XN and IXN	Extraction with methanol-formic acid (99:1, *v*/*v*) + ultrasonication	HPLC-DAD Mobile phase: (A) 1% formic acid in water and (B) acetonitrile Column: Nucleosil C18 (250 × 4.6 mm, 5 µm) λ 286, 370 nm HPLC-ESI-MS/MS (positive mode) Mobile phase: (A) 1% formic acid in water and (B) acetonitrile Column: Nucleosil C18 (125 × 4.6 mm, 3 µm)	[15]
Czech beers and hop extracts	XN, IXN and 8-PN	Hop extracts were extracted with acetonitrile: water (90:10, *v*/*v*) And with supercritical CO_2_ and with supercritical CO_2_ with ethanol as modifier Beer samples were analyzed directly without any treatment	HPLC-APCI-MS (positive and negative modes) Mobile phase: (A) 0.3% formic acid in water and (B) 0.3% formic acid in acetonitrile Column: Purospher Star RP-8e (250 × 4 mm, 5 µm) HPLC-UV λ 330 nm	[16]
Beer	XN	Cloud point extraction (CPE) (Nonionic surfactant Triton X-114; 2.5% of Triton X-114 (*v*/*v*), pH 5, 15% of sodium chloride (*w*/*v*), Equilibrium Temperature 70 °C, Equilibrium time 10 min)	HPLC-UV Mobile phase: MeOH: 0.5% acetic acid in water (80:20 *v*/*v*) Column: ZORBAX Bonus-RP C18 (250 × 4.6 mm, 5 µm) λ 370 nm	[17]
Beer	XN, IXN and 8-PN	Solid-phase Extraction (SPE) (Sep-Pak cartridge)	HPLC-DAD Mobile phase: (A) 1% acetic in acid acetonitrile (B) 1% acetic acid in water and (C) 1% acetic in acid in methanol Column: ODS2 Spherisorb C18 80 Å (250 × 4.0 mm, 5 µm) λ 370, 280 nm	[18]
Biological samples (Samples of urine)	XN, IXN and 8-PN	Solid-phase Extraction (SPE) (SPE cartridges Oasis MCX 96-well plates 60 μm (30 mg))	LC−ESI-MS/MS (negative mode) Mobile phase: (A) 5 mM ammonium bicarbonate buffer pH 7.0 and (B) acetonitrile-methanol (1:1, *v*/*v*) Column: Phenomenex Luna C18 (50 × 2.0 mm, 5 µm)	[19]
Surplus yeast	XN, IXN and 8-PN	Extraction with 95% ethanol + ultrasonic extraction	HPLC-ESI-MS/MS (positive mode) Mobile phase: (A) Water with phosphoric acid pH 1.6 and (B) acetonitrile Column: C18 HyPURITY (150 × 4.6 mm, 5 µm) HPLC-DAD λ 314 nm	[20]
Hops and beers	XN, IXN, 8-PN, 6-PN, desmethylxanthohumol, 6-geranylnaringenin	Hops were extracted with methanol + sonication and beers were diluted with diluted with ethanol–water (5:95, *v*/*v*)	HPLC-APCI-MS/MS (positive mode) Mobile phase: (A) 1% formic acid in water and (B) acetonitrile	[21]
Human serum	XN, IXN, 8-PN and 6-PN	Liquid-liquid extraction (LLE) with methyl-t-butyl ether, followed by an evaporation to dryness and reconstitution the residue in 70% methanol	UHPLC-ESI-MS/MS (negative mode) Mobile phase: (A) 0.1% formic acid in water (B) acetonitrile Column: Shim-pack XR-ODS III C18 (50 × 2.0 mm, 1.6 µm)	[22]
Hop pellets	XN	High-pressure treatment 250 MPa/5 min + 200 MPa/5 min +300 MPa/5 min	HPLC-UV/VIS Mobile phase: (A) 1% formic acid in water (B) acetonitrile Column: Waters (Milford, MA) RP C18 (250 × 4.6 mm, 5 µm) λ 370 nm	[23]

XN: xanthohumol; IXN: isoxanthohumol; 8-PN: 8-prenylnaringenin; 6-PN: 6-prenylnaringenin; HPLC-DAD: High-Performance Liquid Chromatography coupled with Diode-Array Detection; UHPLC-PDA: Ultra- High-Performance Liquid Chromatographic coupled with PhotoDiode Array; HPLC-ESI-MS/MS: High Performance Liquid Chromatography coupled with ElectroSpray Ionisation Tandem Mass Spectroscopy; HPLC-APCI-MS: High Performance Liquid Chromatography coupled with on-line Atmospheric Pressure Chemical Ionization Mass Spectrometry; HPLC-UV: High-Performance Liquid Chromatography coupled with Ultraviolet detector; LC−ESI-MS/MS: Liquid Chromatography coupled with ElectroSpray Ionisation Tandem Mass Spectroscopy.

**Table 2:** Description of the composition and analytical concentration of XN detected in food supplements (A, D, G, M and N) and hops (H).

Code	Health Claims	Declared Hops Extract Content (mg/unit)	Analytical Contents of XN (µg/unit) *	Dose Form and Intake Recommendation	Other Ingredients
A	Stimulates a normal state of mind, relaxation and mental well-being	150	142.6 ± 4.884	2 capsules/day	Standardized extracts of griffonia, l-tryptophan, Vitamin B_6_, Vitamin B_1_, Vitamin B_9_, Vitamin B_12_, magnesium stearate and vegetable gelatin
D	Temporary and light states of nervousness and occasional difficulty in falling asleep	27.8	56.42 ± 3.403	Nervousness: take 1 or 2 capsules 1 to 3 times every 24 h. Max (6 × 139 = 834 mg) To sleep: take 1 or 2 capsules half an hour before bedtime, and if necessary, take an equal dose in the afternoon. Max (4 × 139 = 556 mg)	Dry methanol extract 45% *v*/*v* of *Valeriana officinalis* root, hydrogenated and partially hydrogenated soybean oil, lactose monohydrate, sorbitol solution (not crystallizable) and E 422
G	Helps to sleep. Irritability, mood swings, hot flashes	100	<LOD	1 capsule/day for three months	Soy isoflavones, salvia officinalis, evening primrose oil, magnesium silicate, vitamin E, magnesium stearate, colloidal silica, Vitamin B_6_, Vitamin D_3_
M	Discomfort associated with menopause such as hot flashes, sweating, restlessness and irrationality	100	3602 ± 72.47	1 capsule/day	Bitter acids, prenylated flavonoids, gelatin, silicon dioxide, Vitamin B_6_
N	Helps to fight the signs derived from stress	30	57.51 ± 1.318	1 capsule/day	*Valeriana officinalis*l extract, *Passiflora incarnata* l extract, *Tilia argentea* l extract, l-tryptophan, magnesium, Vitamin B_6_, Vitamin B_5_, E-470b, E-460, E-551, E-171, gelatin, calcium pantothenate, pyridoxine hydrochloride
			Analytical contents of XN (µg/100 mg of hops) *		
H	Contributes to the balance of the organic functions	n.a.	85.57 ± 10.86	1 spoon (~100 mg)/cup of water for infusion preparation	n.a.

* Data expressed as mean values ± standard deviation (*n* = 3). n.a., not applicable.

**Table 3:** Description of commercial beers samples and concentration of XN detected.

Code	Type of Beer	Container Vol. (cc)	Alcohol Degree (%)	Analytical Contents of XN (µg/L) *	Analytical Contents of XN (µg/can or Bottle) *	Other Ingredients
B	Craft	33	5.0	28.49 ± 1.593	9.403 ± 0.5255	Water, barley malt, hops and yeast
E	Lager	33	5.5	61.57 ± 3.300	20.32 ± 1.089	Water, barley malt, maize and hops
Mh	Amber	33	7.5	29.82 ± 1.268	9.840 ± 0.4185	Water, barley malt, maize and hops
MS	Blonde no alcohol	33	<1	<LOD		Water, barley malt, maize, hops and E330
S	Stout	44	4.2	<LOD		Water, malt, barley, roasted barley, hops and azote

* Data expressed as mean values ± standard deviation (*n* = 3).

**Table 4:** Retention time, precision, linearity and sensitivity parameters of the HPLC-diode array detector (DAD) method for XN analysis.

Compound	t_R_ (min)	RSD (%)	λ_max_ (nm)	Intercept	Slope	*r* ^2^	Range (mg/L)	LOD (mg/L)	LOQ (mg/L)	Intra-Day RSD (%)	Inter-Day RSD (%)
Xanthohumol	10.03	0.07	370	−1.949 ± 3.371	226.8 ± 1.267	0.9999	0.05–20	0.016	0.049	1.05	1.83

t_R_, Retention time; LOD, limit of detection; LOQ, limit of quantification; RSD, relative standard deviation.

**Table 5:** Accuracy and precision of the Ultrasound (US)-assisted extraction-HPLC-DAD method for hops and food supplements at three concentration levels of XN (*n* = 3).

Samples	XN ^a^		Spiked Level of XN					
			98 µg/g		245 µg/g		490 µg/g	
	Original Content	Precision Intra (RSD)	Accuracy (Recovery)	Precision Intra (RSD)	Accuracy (Recovery)	Precision Intra (RSD)	Accuracy (Recovery)	Precision Intra (RSD)
	(µg/g)	(%)	(%)	(%)	(%)	(%)	(%)	(%)
A	268.96	3.43	72.55	4.30	102.5	2.03	101.5	3.53
D	106.45	6.03	94.77	13.6	83.43	19.6	108.2	6.43
G	<LOD		100.9	0.632	100.7	1.30	104.0	1.28
N	117.38	2.69	109.6	6.79	103.8	0.26	105.0	1.00
			9800 µg/g		14,700 µg/g		19,600 µg/g	
			Accuracy (Recovery)	Precision Intra (RSD)	Accuracy (Recovery)	Precision Intra (RSD)	Accuracy (Recovery)	Precision Intra (RSD)
			(%)	(%)	(%)	(%)	(%)	(%)
M	12640	2.01	96.78	15.0	86.01	10.3	90.28	1.10
			980 µg/g		2450 µg/g		4900 µg/g	
			Accuracy (Recovery)	Precision Intra (RSD)	Accuracy (Recovery)	Precision Intra (RSD)	Accuracy (Recovery)	Precision Intra (RSD)
			(%)	(%)	(%)	(%)	(%)	(%)
H	855.68	13.0	84.65	3.98	88.94	1.20	76.37	3.25

^a^ Content of XN determined in hops and food supplements tested as the mean values of three replicates ± standard deviation and expressed as µg/g.

**Table 6:** Recoveries and precision of the solid-phase extraction (SPE)-HPLC-DAD method in different commercial beers at three concentration levels of XN (*n* = 3).

	XN ^a^		Spiked Level of XN					
			49 µg/L		98 µg/L		490 µg/L	
	Original Content	Precision Intra (RSD)	Accuracy (Recovery)	Precision Intra (RSD)	Accuracy (Recovery)	Precision Intra (RSD)	Accuracy (Recovery)	Precision Intra (RSD)
	(µg/L)	(%)	(%)	(%)	(%)	(%)	(%)	(%)
B	28.49	5.59	78.67	15.0	79.81	7.06	73.30	5.18
E	61.57	5.40	106.9	18.1	109.6	8.00	104.7	0.599
Mh	29.82	4.25	86.63	4.02	97.22	34.7	88.31	16.1

^a^ Content of XN determined in different commercial beers tested as the mean values of three replicates ± standard deviation and expressed as µg/L.

**Table 7:** Retention time and MS/MS parameters for the identification of XN in negative mode.

Molecule	t_R_ (min)	Precursor Ion (*m*/*z*)	Product Ions (*m*/*z*)	Collision Energy (V)
Xanthohumol (XN)	8.27	353.2	119.1	−37
			174.9	−35
			233.0	−22

t_R_, Retention time.
